# IoT-based data-driven predictive maintenance relying on fuzzy system and artificial neural networks

**DOI:** 10.1038/s41598-023-38887-z

**Published:** 2023-07-27

**Authors:** Ashraf Aboshosha, Ayman Haggag, Neseem George, Hisham A. Hamad

**Affiliations:** 1 Rad. Eng. Dept., NCRRT, Egyptian Atomic Energy Authority (EAEA), Cairo, Egypt; 2 Electronics Technology Department, Faculty of Technology and Education, Helwan University, Cairo, Egypt

**Keywords:** Energy science and technology, Engineering

## Abstract

Industry 4.0 technologies need to plan reactive and Preventive Maintenance (PM) strategies for their production lines. This applied research study aims to employ the Predictive Maintenance (PdM) technology with advanced automation technologies to counter all expected maintenance problems. Moreover, the deep learning based AI is employed to interpret the alarming patterns into real faults by which the system minimizes the human based fault recognition errors. The Sensors Information Modeling (SIM) and the Internet of Things (IoT) have the potential to improve the efficiency of industrial production machines maintenance management. This research work provides a better maintenance strategy by utilizing a data-driven predictive maintenance planning framework based on our proposed SIM and IoT technologies. To verify the feasibility of our approach, the proposed framework is applied practically on a corrugated cardboard production factory in real industrial environment. The Fuzzy Logic System (FLS) is utilized to achieve the AI based PM while the Deep Learning (DL) is applied for the alarming and fault diagnosis in case the fault already occured.

## Introduction

Industry 4.0 technology is a revolution of the way factories achieve mass production, improve, and distribute their products. Producers are merging new techniques, including IoT, cloud computing and computational analytics with AI and machine learning into their production lines during the manufacturing process. Through the continuous development of AI based automation technologies, the IoT and their applications have the opportunity to benefit from the industrial development, especially in applying PdM systems^1^. Through high precision smart sensors, we collect a huge amount of data that indicate the state of machines and their healthy or faulty operation^2^. By analyzing these data, we predict quickly and accurately the possible failures of the machine. PdM strategies reduce machine maintenance costs and extend the service lifespan of machine parts. The probability of line production machine faults are calculated on the basis of historic data from previously tested the expert systems^3^. Machine parameters (such as machine type), operator experience, machine age, operation time, component types and operation location), are taken into consideration for fault diagnosis and prediction. This research work discusses the role of the AI based IoT and its development, by integrating heterogeneous distributed sensor data of all machines to analyze their operation conditions^4^. To achieve this target, we developed an algorithm that helps analyze the data and to make a precise decision^5^. The presented research work aims to prevent halting the production line without reasonable causes.

### Benefits of predictive maintenance

Relying on predictive maintenance, it is possible to improve the performance of production machines, predict their conditions and recognize faults, predict their maintenance dates, and also predict their lifetime^6^ based on analyzing and processing the collected data. When a faulty operation takes place by an operator, the system analyzes the performance, and the operator can be quickly notified of the faulty act. By applying the proposed system, it is expected to avoid breakdowns, reduce loss of time, minimize the effort, limit the operation cost^7^, and predict failure before its occurrence. The most important results are to increase the operating lifetime of machines, reduce the cost of spare parts, reduce production downtime, improve product quality, and save energy.

### Causes behind machine failure

The overall production line depends on the machinery and a slight failure may cause the entire production line to halt. Whether it is a failure in a sub or auxiliary parts, the cause of the failure may be on behalf of the operators. In the long run, if the basic parts of the machines in the wrong operation continues, failure will follow after the other, which will increase the cost and consume a longer time. This faulty operation will negatively affects the production process^8^. Due to this faulty operation the percentage of wastage in production will also increase, and the failure could be due to the natural malfunctions of the machine that depend on the lifetime of the spare parts. This research work will lead to precisely recognition of PM and predicts faults clearly before the malfunction occurs^9^.

### Research motivations

#### Research motivations

The needs arose in this study are to determine severity and risk evaluation of sudden failure of corrugated cardboard production machine parts which may lead to prolong the wasted time. Generally, PM has to be carried out every week for inspection, revision and lubrication of mechanical parts, every month to remove the mechanical and electrical parts that have been monitored for abnormal performance which require close inspection before unexpected failures occur^10–12^. This research aims to determine the sensors on the machine which recognize failures before they occur.

The rest of the paper is organized as follows; “Related works” section presents the related works. “The proposed predictive maintenance system” section introduces the proposed predictive maintenance system. “Implementation of the proposed system” section explains applying proposed system. “Methodology” section is the methodology. “Failure mode analysis methodology” section presents Failure Mode Analysis Methodology. “Correlation analysis” section presents Correlation Analysis. “Platform and data analysis” section presents platform data analysis. “Conclusion” section is the discussion and results analysis.

## Related works

Industry 4.0 technologies development and progress result in rapid increase in the size of machines, quantity of production modules, mass production, mega stores, big data, and high precision. These changes require for sure the IoT, AI and High Performance Computing (HPC) to apply the predictive maintenance. This motivated many researchers recently to direct their research studies to the field of data analysis for predictive maintenance which became an essential tool in industry 4.0 Technology. Rabatelab et al.^13^ extended the anomaly detection problems. To extract the patterns, they developed a method to considering contextual parameters associated with data. Then compliance of the new data and, according to the knowledge extracted, can detect anomaly. Zhang et al.^11^, developed a method to reveal the temporal-dependency embedded in sensor data streams as the basis for engine degradation prediction. Huang et al.

Reference^14^ showed that machine maintenance is ramified issue that relates to many other factors for the requirements of modern industrial development. When symmetric components do not perform the same function, the system is out of balance. A preventive maintenance action is performed once the state difference. The research did not take into account the difference in method of operation despite the similarity of the elements. Wanga and Miaob^15^ presents Predictive maintenance for aircraft systems relying on proportional hazard models. The authors link open overlay panel proposed by Wim et al.^16^ showing that aircraft operating in hot, sandy airports or regions have very different circumstances of operation than aircraft operation in normal cold, wet airports, will lead to several failure modes and lifetimes for spare parts. Aircraft fault prediction at maintenance service on the basis of regression data and aircraft parameters are studied by Pogačnik et al.^17^ for predictive Maintenance and Reliability. Brauers and Balezentis^18^ developed a Multi-Objective Decision Making technique for Linguistic Reasoning with an Application to staff Selection. The FMEA Traditional Modifications (FMEA Improvement) in IT Risk management is proposed by Nina Fadilah et al.^19^. Liu et al.^20^ studied the Failure mode and its effect analysis.

In 2020, Mourtzis^21^, this paper investigates the major historical milestones in the evolution of manufacturing systems simulation technologies and examines recent industrial and research approaches in key fields of manufacturing and PM. In 2021, Filz et al.^22^, proposed a data-driven Failure Mode and Effect Analysis (FMEA) methodology by using deep learning models on historical and operational data from the use stage of industrial investment goods. The developed methodology is supposed to support the maintenance planning for industrial investment goods by enhancing transparency and providing decision support. In 2021, Mourtzis et al.^23^, presented a Computer Aided Design (CAD) and Computer Aided Manufacturing (CAM) can be considered as the cornerstones of the lifecycle of a manufacturing asset. Since the above-mentioned processes often involve multiple engineers, from different departments even from different companies, it is crucial to ensure the flawless communication between the different individuals as well as to make the design process more intuitive. In 2018, Mourtzis et al.^24^, proposed a framework for the modeling of milling and lathe CNC machine-tools, through a general machine model. The framework is based on the Open Platform Communications—Unified Architecture (OPC-UA) communications standard to provide a macroscopic and microscopic view of machine shops, towards the Machine Shop 4.0 concept.

In 2021, Martins et al.^25^, in this paper a methodology to build monitoring solutions for machining devices is defined, based on the main equipment and operations used by molds industry companies. For a standardized approach, OPC-UA is used for high-level communication between the various systems. In 2019, Liu et al.^26^, this paper proposes a CPMT Platform based on OPC-UA and MT Connect that enables standardized, interoperable and efficient data communication among machine tools and various types of software applications. In 2021, Schmid et al.^27^, proposed Acquisition of machine tool data via the open source implementation open62541 for OPC-UA. current Industry 4.0 Communication methods are defined as OPC-UA, MQTT, or REST. These communication standards can integrate machine tool and process data in IoT-Clouds or external devices. In 2022, Eswaran and Bahubalendruni^28^, this paper demonstrates the research progress and developments in the AR/VR technologies for product design and evaluation, Repair & Maintenance, Assembly, Warehouse management, Quality control, Plant layout and CNC Simulation. The critical challenges in AR/VR technology implementation at hardware and software level and opportunities for the further developments are discussed.

In 2022, Xiong et al.^29^, presented the concept of intelligent additive manufacturing and design (IAMD) while providing a triple-layer model for reference. Details about these three layers, i.e., digital thread layer, cyber-physical layer, and intelligent service layer, are presented. Moreover, both scientific and engineering challenges rose during the studies and implementations of IAMD are discussed together with potential solutions. In 2023, Liu et al.^30^, in this study, we develop a novel and highly practical maintenance model to fill the gap. The proposed model simultaneously considers the predictive maintenance and inventory policies of spare parts. In light of the high degree of complexity in modern manufacturing systems and the profound stochastic city in component degradation, a met modeling-based simulation optimization method is proposed to find the optimal component inventory policy and degradation level thresholds of each component. In 2022, van Dinter et al.^31^, this research aims to gather and synthesize the studies that focus on predictive maintenance using Digital Twins to pave the way for further research. A systematic literature review (SLR) using an active learning tool is conducted on published primary studies on predictive maintenance using Digital Twins, in which 42 primary studies have been analyzed. In 2023, Gupta et al.^32^, this paper presents a scalable and economical maintenance 4.0 solution for such a system using data from sensors installed (on a live system in absence of historical data). Differentiating between anomaly detection and outlier detection the paper presents an algorithm that can be used to remove idle and noisy data from the datasets. In 2022, Liu et al.^33^, this article proposed Predictive Maintenance (PdM), as a pivotal part of Prognostics and Health Management (PHM), plays a vital role in enhancing the reliability of machine tools in the Internet of Things (IoT)-enabled manufacturing. In order to realize a highly reliable maintenance plan integrated with the fault prediction, the maintenance decision-making, and the Augmented Reality (AR)-enabled auxiliary maintenance, an intelligent predictive maintenance approach for machine tools is proposed in this paper via multiple services cooperating within a single framework.

## The proposed predictive maintenance system

In this research work we study the possibilities of applying the human faulty operation effect on PdM. Data are collected and analyzed to identify operational measurable factors and its influence on the maintenance. We adopt a PdM strategy based on the availability of performance over time, service life prediction models, and Methods for developing a decision support system for corrective maintenance, PdM. Basis of maintenance, and monitoring system. Design of a machine is depending on the technologies of the current industrial revolution, and production lines system’s needs^10^. The first important step is to activate the role of IoT applications^4^.

### Data acquisition system

**Figure 1:**
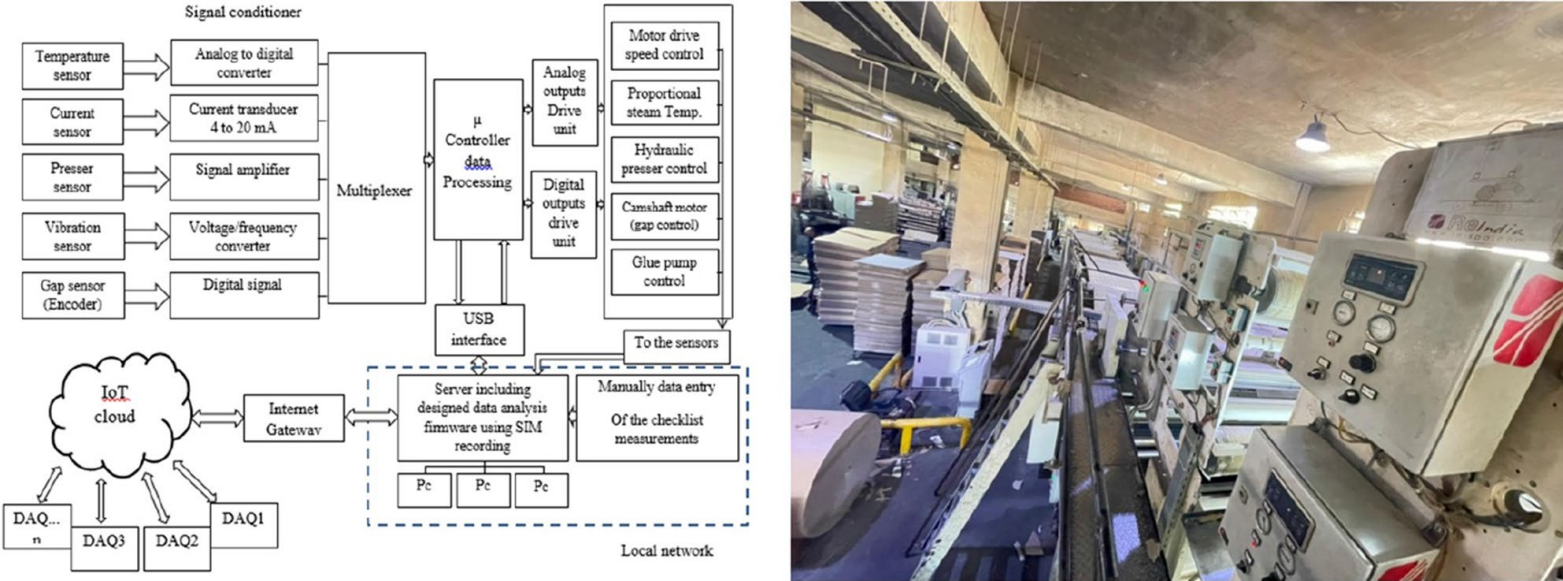
Data acquisition system (DAQ) architectural overview on the left side and the overall corrugated cardboard factory on the right side.

The data acquisition system is used to collect the output signals of sensors located on all parts of the production lines and converts them into addressable electronic signals and digital data that can be analyzed and compared to increase data integrity and to provide an analysis of these data for the benefit of the production process. Figure 1 shows an architectural overview of the data acquisition system and the IoT interface diagram of the corrugated cardboard factory where this research study is applied practically.

### Data aggregation

All of sensors are connected to an electronic interface card (velamen interface card type: k8055) that is connected to the internal network of the factory and then to the cloud through the Internet gateway using Message Queuing Telemetry Transport (MQTT) protocol to connect to the IoT such as shown in Fig. 1. For more about IoT Interface and Communication, see^11^.

## Implementation of the proposed system

To assess the effectiveness of the proposed system, we implemented it on a corrugated board factory in Egypt as a case of study. The following subsections present the implementation of the proposed system to apply our proposed approach in a real case of study.

**Figure 2:**
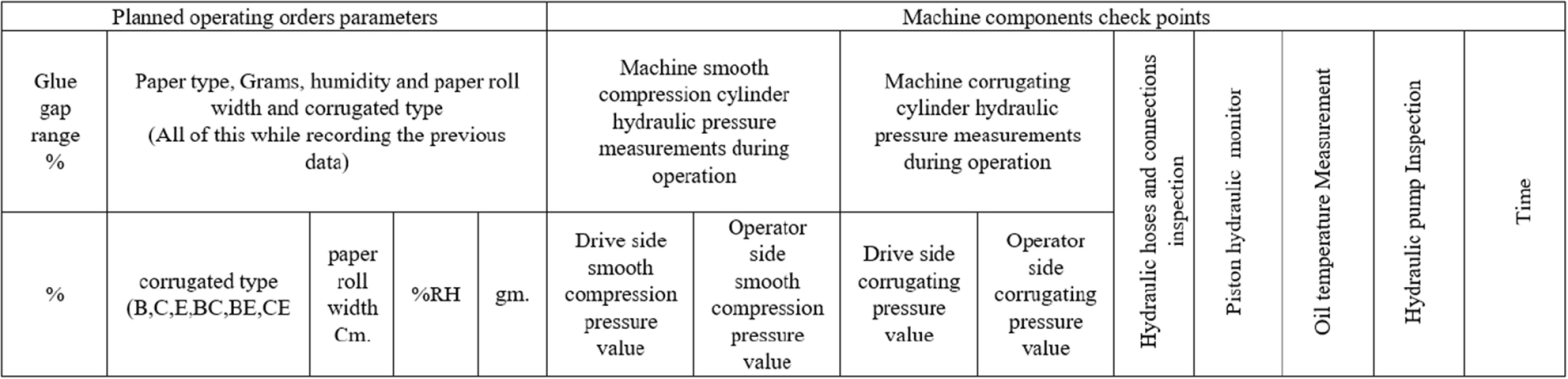
Implementation of the proposed system.

### Machine data acquisition and operation monitoring

Monitoring of faulty operation in the hydraulic systems of the machine maintenance in corrugating and gluing paper layers machine is depending on the operation orders. The monitoring of a human operation is depending on the recommended operation order requirements^2^. Data of this form are recorded for each running order as shown in Fig. 2.

### Determination of machine sensors status

**Figure 3:**
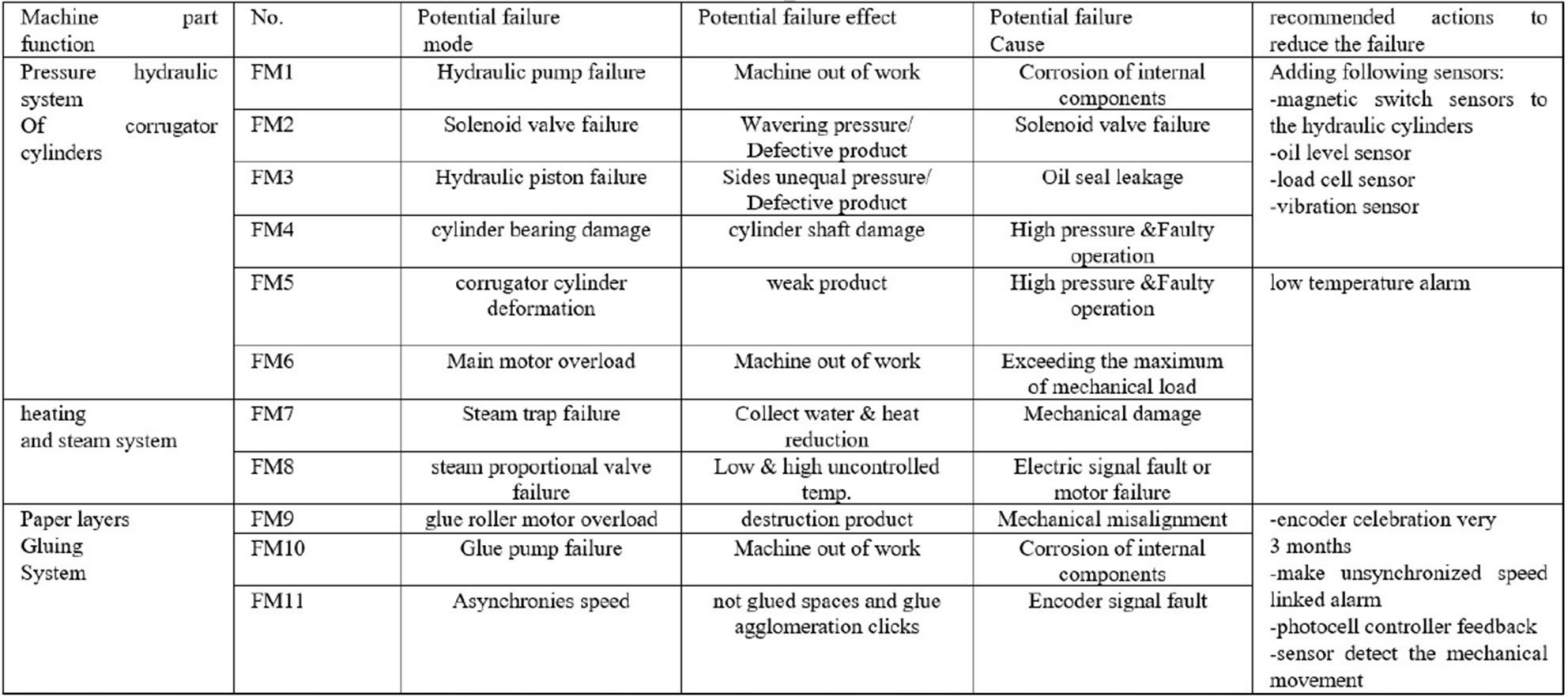
The FMEA of the corrugator and glue machine.

Appropriate exploration method, an active scan method, is applied to identify which parts and components of the machine need to have more sensors to provide the system with sufficient data. To achieve this target, we rely on applying the information Failure Modes and Effect Analysis (FMEA), Fig. 3, based on continuous measurements recorded of the sensors data acquisition system. We can plan and apply a Proactive Risk Analysis (PRA) model to sets values from 1 to 10 for each of the factors Severity, Occurrence, and Detection, for each component in the machine to get the Rick Priority Number (RPN) that represents the extent of the hazard and its location in the machine. The Benefit of the RPN values is to know the hazard components in the machine that require to get more sensors by which that makes the detection process easier. Contineously, the system collects the faults data and compare them to the value of the RPN within the previous values and it monitors the difference.

### IoT protocols compared: MQTT vs. OPC-UA

In IoT world, the challenges of system networking are more clear than ever. The introduction of digital technologies is becoming increasingly relevant. IoT applications range from Big-Data approach to full digital Industrial IoT solutions. These applications need not only a new technology in terms of IoT networks, but also innovative methods to integrate existing machines and AI based IoT workflows. In the arena of industrial communication protocols, two standards dominate this context: The first is the OPC-UA andthe second is the MQTT, which are compared in this article.

#### Functionality differences

##### MQTT architecture

The MQTT message protocol relies on the concept of the publish-subscribe pattern. Important for the publish-subscribe model of MQTT is a broker, which is the key point of communication. Clients can be interfaced to the message broker and handle data with each other through topics, which are sent with messages. If a message is published on a certain topic, then this is transferred to all participants with subscription of this topic.

##### OPC-UA architecture

The server/client design is the classical communication protocol in OPC-UA. It relies on the idea that there is a passive server component that delivers data for all client applications in the system. These applications may access data from the server through several standard services.

#### Typical standard applications

##### Application cases of MQTT

IoT applications are the primary utility case for MQTT. When data are needed from remote stations and sent over an unreliable internet, MQTT should be the first choice. MQTT can guarantee secure and complete data exchange through built-in properties. Another typical use case of the messaging protocol is the IoT gateways that integrate sensor data, pre-process it, and send it to the cloud for data analysis via MQTT.

##### Application cases of OPC-UA

The main case of the OPC-UA communication protocol is the closed-loop system control in the local area network (LAN). When machines on a factory floor require to communicate in real time, the standard presents its strengths. The standard is especially suitable for the integrated services and extensions of SCADA systems.

## Methodology

In the methodology we assess the failure impact and apply Fuzzy MULTIMOORA method.

### Preventing failures by the assessment of impacts

**Figure 4:**
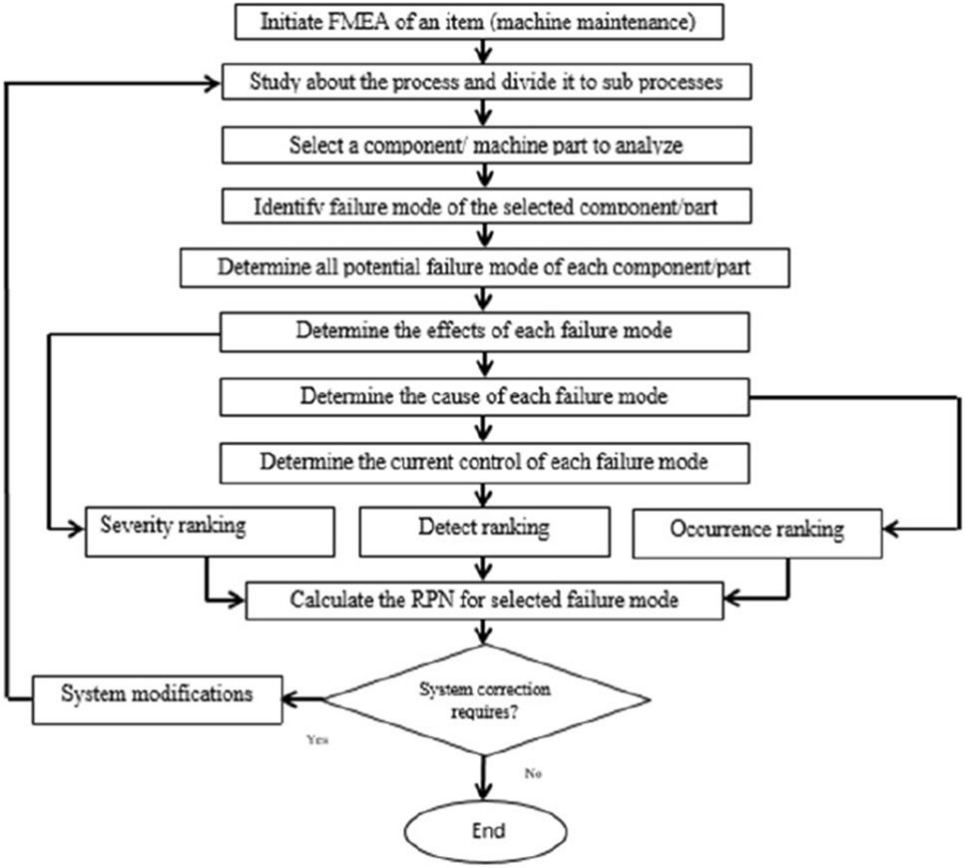
The traditional FMEA procedure.

We use FMEA which is a methodology that aimes to prevent failures relying on the perfect assessment of impacts, and then determines the priority of possible actions for reducing the occurrence of failures. Applying of FMEA is achieved to the maintenance system where the analysis of FMEA distinguishes the possible failure modes, evaluates the cause and effects of dissimilar failure modes, and predict of high-risk failures^19^. Conventional FMEA makes use of a Risk Priority Number (RPN) to calculate failure elements *O*, *SandD*, where *O* is the probability occurrence of the failure, *S* is the severity of the failure, and *D* is the ability of detection for each failure. Each of risk elements have mathematical scale from 1 to 10. The risk priority is directly proportional to the RPN of the failure mode. Figure 4 shows traditional FMEA method flowchart.

### Fuzzy MULTIMOORA method

**Figure 5:**
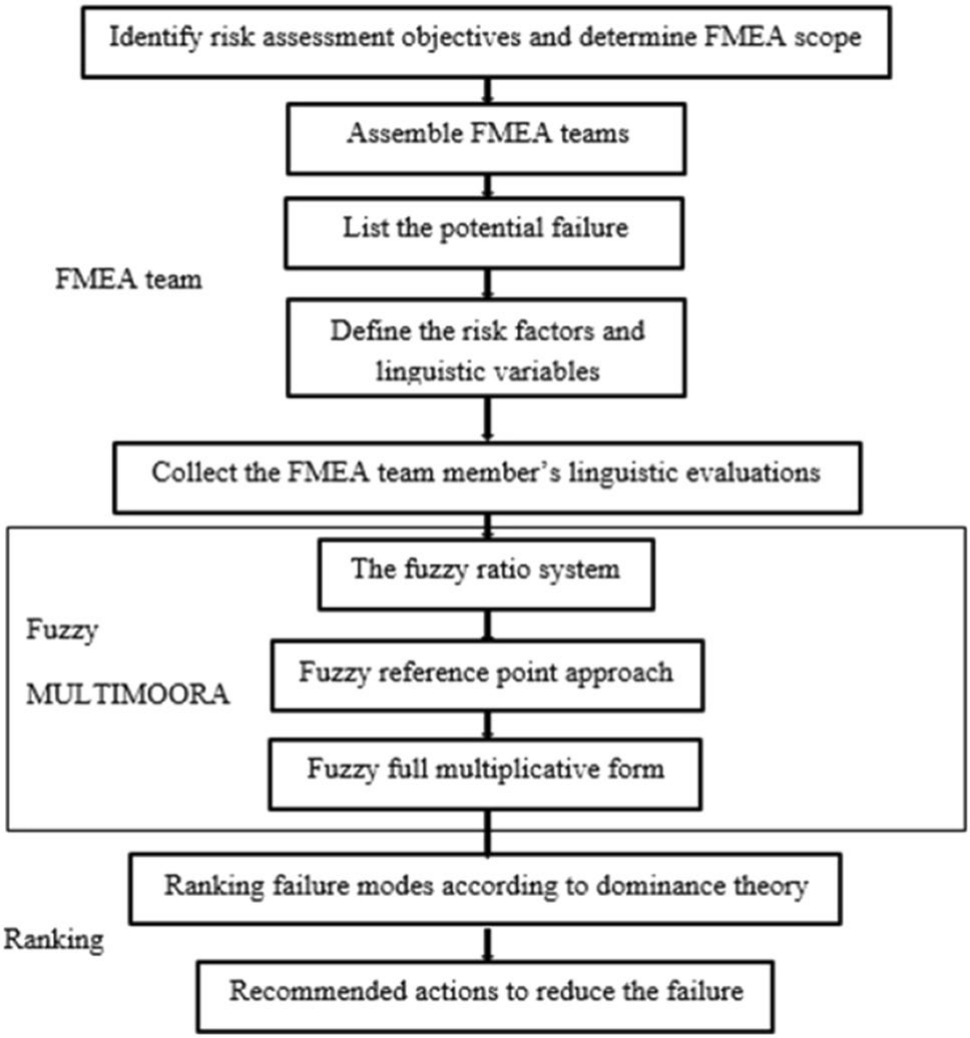
The traditional ranking identifies failure modes procedure.

FMEA is a methodology uses the potential failure modes to prevent machine failures depending on the evaluation of impacts, probability of occurrence, and how difficult is it to detect. After that, FMEA determines the priority of possible actions to eliminate the occurrence of this failures. FMEA helps to study the influence of machine parts failures on system performance, safety, and machine functions success. The multi-objective optimization by ratio analysis (MULTIMOORA) method is a recently introduced based on the multi-criteria decision-making (MCDM) methods^34,35^. These are considered the best way to analyze the potential failure modes, their causes, and arrange them depending on the priority and the effects of failure on the machine. This mainly aims to assess the risk associated with the identified failure modes and prioritize them. The main target of this is to increase the ability of the system to predict the failure in any machine part to support expansion of the scope of its industrial applications. MOORA is the main idea of MULTIMOORA method. MCDM technique makes use of a new model where fuzzy set concept and MULTIMOORA method are implemented for the evaluation of failure modes and rating in FMEA^36,37^. The risk factors and their relative weights are treated as fuzzy variables and evaluated by using fuzzy linguistic terms using different teams and fuzzy ratings^38^. MULTIMOORA method is used mainly to compare and to determine the real risk ranking of the failure modes that have been identified and calculate the effect of its weights. We study the trapezoidal fuzzy numbers which will be utilized in the FMEA model proposed in this article. Figure 5 shows the flowchart diagram of rank identify failure modes using MULTIMOORA method in FMEA process^39^.

### Applied case of study

A case of study of corrugated cardboard machine is provided to illustrate the practicality and usefulness of the proposed Fuzzy FMEA approach. The main purpose of that is to arrange all failures and determine the extent of the risk exposure by which we carry out the appropriate maintenance procedure^38–40^. Ensuring the non-stop production requires a solid maintenance plan. Our machine consists of three major systems hydraulic, steam, and gluing where it is intended to conduct an FMEA project to minimize the potential for failure occurrence. Through the data collection and the sensors readings, 11 potential failure modes were explored and listed, presented in Fig. 3, the FMEA of the corrugator & glue machine. These failure modes have risk factors (*S*, *O*, *andD*), to calculate RPN for each failure mode which are presented in Table 1.

**Table 1:** FMEA (risk priority number) RPN ratings.

FM	Failure mode	Severity(S)	Occurrence(O)	Detection(D)	RNP	Ranking
FM1	Hydraulic pump failure	9	2	1	18	11
FM2	Solenoid valve failure	8	3	4	96	8
FM3	Hydraulic piston failure	7	4	6	168	4
FM4	Cylinder bearing damage	9	1	8	72	9
FM5	Corrugator cylinder deformation	9	5	3	135	5
FM6	Main motor overload	8	7	2	112	6
FM7	Steam trap failure	6	6	5	180	3
FM8	Steam proportional valve failure	7	4	7	194	2
FM9	Glue roller motor overload	8	3	2	48	10
FM10	Glue pump failure	9	7	4	252	1
FM11	Asynchronies speed	7	2	7	98	7

### Ethics approval

Helwan University and EAEA Research had approved the publishing of this article.

## Failure mode analysis methodology

FMEA team member $MT_{k}(k=1,2,\ldots ,l)$ is responsible for the assessment of *m*.

*Step 1* The objectives of the failure risk assessment are defined and the scope of the FMEA is accurately described. Defining the main machine function and its sub-parts and defining the systems and components that we want to analyze for failure. This step controls the results failure modes $$ FM_{i}(i = 1,2,\ldots m) $$ with respect to n risk factors $$ ({RF}_{J}()j=1, 2, n)$$. Each $$ ({MT}_{k}) $$ is given a weight $$ (\lambda _{k} > 0(k = 1,2,\ldots ,l)) $$ satisfying $$(\sum _{k = 1}^{l}{\lambda _{k} = 1}) $$, Let $$ ({\widetilde{R}}_{k} = ({\widetilde{r}}_{{ij}}^{k})_{m*n}) $$ Fuzzy assessment matrix of the kth team member, where $$(\ {\widetilde{r}}_{{ij}}^{k} = (r_{{ij}1}^{k}),(\ r_{{ij}2}^{k},r_{{ij}3}^{k},r_{{ij}4}^{k}))$$ is the fuzzy rating. Let $$ ({\widetilde{w}}_{j}^{k})=(w_{j1}^{k},w_{j2}^{k}, w_{j3}^{k},w_{j4}^{k}) $$ is the fuzzy weight of the risk factor $$ ({RF}_{J})$$ is given by $$({MT}_{k}) $$. Based on the above we can procedure the risk ranking of the failure modes as the following steps.

*Step 2* To execute the MULTIMOORA method, team 1 and team 2 members (TM1, TM2) have weights (0.55 and 0.45) respectively for the same failure modes FM1 to FM11 RPN calculation. The two teams’ members can rate the failure modes and risk factors in linguistic terms as well. The linguistic ratings given by the two team members are (TM1, TM2).

*Step 3* Define risk factors and select suitable linguistic variables set of risk elements and their calculation metrics which are established to evaluate the hazard of failure modes. FMEA teams members use the linguistic terms to evaluate the importance of risk factors and the rate failure modes. Then, transformed into fuzzy numbers with the correspondence of their fuzzy equivalents.

*Step 4* Assemble the teams’ members linguistic assessment of the fuzzy collected ratings which belong to failure modes of each risk factor. Then, risk factors can be calculated to set the fuzzy group matrix. The data aggregation for risk factors and weighs then can be deducted.

1 $$\begin{aligned} (\widetilde{R}= & {} {(\widetilde{r})}_{m \times n}), \end{aligned}$$

2 $$\begin{aligned} {\widetilde{r}}_{\text {ij}}= & {} \left( r_{\text {ij}1},r_{\text {ij}2},r_{\text {ij}3},r_{\text {ij}4} \right) = \left( \sum _{k = 1}^{i}{\lambda _{k}r_{\text {ij}1}^{k},}\lambda _{k}r_{\text {ij}2}^{k},\lambda _{k}r_{\text {ij}3}^{k},\lambda _{k}r_{\text {ij}4}^{k} \right) . \end{aligned}$$

Aggregated fuzzy weight for each risk factor ${\widetilde{w}}_{j}$ is calculated as

3 $$\begin{aligned} {\widetilde{w}}_{j} = \left( w_{j1},w_{j2},w_{j3},w_{j4} \right) = \left( \sum _{k = 1}^{i}{\lambda _{k}w_{j1}^{k},}\lambda _{k}w_{j2}^{k},\lambda _{k}w_{j3}^{k},\lambda _{k}w_{j4}^{k} \right) . \end{aligned}$$

*Step 5* The Normalized fuzzy assessment matrix uses the fuzzy ratio system to clear the normalization of the fuzzy numbers according to appropriate values of fuzzy numbers. The aggregated fuzzy values are calculated by Eqs. (4) and (5) are used to calculate $\widehat{r}$. The normalized fuzzy assessment matrix is then presented.

4 $$ \left( {x_{{{\text{ij}}}}  = \left( {x_{{{\text{ij}}1}} ,x_{{{\text{ij}}2}} ,x_{{{\text{ij}}3}} ,x_{{{\text{ij}}4}} } \right) = \left( {w_{{j1}} \frac{{r_{{{\text{ij}}1}} }}{{\hat{r}}},w_{{j2}} \frac{{r_{{{\text{ij}}2}} }}{{\hat{r}}},w_{{j3}} \frac{{r_{{{\text{ij}}3}} }}{{\hat{r}}},w_{{j4}} \frac{{r_{{{\text{ij}}4}} }}{{\hat{r}}}} \right)} \right)\quad \left( {\hat{r} = \sqrt {\sum\limits_{{i = 1}}^{m} {r_{{{\text{ij}}4}}^{2} } } } \right). $$

The ratio $({\widetilde{y}}_{i})$ for each failure mode can be calculated by the following Eq. (5):

5 $$\begin{aligned} \left({\widetilde{y}}_{i} = \sum _{j = 1}^{g}{{\widetilde{x}}_{\text {ij}} -}\sum _{j = g + 1}^{n}{\widetilde{x}}_{\text {ij}}\right), \end{aligned}$$

where $g = 1, 2\ldots {} n$ stands for number of factors to be minimized. Failure modes with higher defuzzified values $(\overline{y}\text {i\ })$ are attributed to higher ranks.

*Step 6* The fuzzy maximal objective reference point (MORP) vector:

$({\widetilde{r}}^{*} = ({\widetilde{x}}_{1}^{*},{\widetilde{x}}_{2}^{*},{\widetilde{x}}_{3}^{*},{\widetilde{x}}_{4}^{*}))$ is obtained according to the matrix $(\widetilde{X} = [{\widetilde{x}}_{\text {ij}}]_{m*n})$.

The distance of each failure mode from the fuzzy MORP can be calculated the appropriate ranking. The ranking orders of all failure modes are defined according to the eccentricity from the reference point, Eq. (6).

6 $$\begin{aligned} (d_{i = \max {d\ (}}{\widetilde{x}}_{j}^{*},\ {\widetilde{x}}_{\text {ij}})). \end{aligned}$$

*Step 7* The fuzzy full multiplicative of the overall utility, the *ith* failure mode, can be expressed as dimensionless fuzzy number by Eq. (7).

7 $$\begin{aligned} (\widetilde{U} = \ {\widetilde{A}}_{i} \oslash \ {\widetilde{B}}_{i}), \end{aligned}$$

where $({\widetilde{A}}_{i} = \prod _{j = 1}^{g}{\widetilde{x}}_{\text {ij}}\ )$ The product of factors of the ith failure mode to be minimized and $({\widetilde{B}}_{i} = \prod _{j = g + 1}^{n}{\widetilde{x}}_{\text {ij}})$ is the product of factors of the ith failure mode, to be maximized. Then utility $(\widetilde{U})$ is transformed into crisp values $(\overline{U})$, the higher $({\overline{U}}_{i})$ is the higher the rank of certain failure mode. The fuzzy full multiplicative higher ranks are then presented and the ranking of failure modes lists, derived from the three previous steps, are calculated.

## Correlation analysis

Correlation analysis is one of the most important outputs of this research which is applied to analyze data and identify correlation and regression analysis of the variables. In this case of study, the effect of faulty operation with the failure modes (shown in Fig. 3) in this section, the traits, and correlation analysis were explored. We examined the relationships between the variables represented in the sensor data and the impact of the faulty operation factor to clarify their effect to the possibility of machine failure on the performance of the production process.

*r* represents the correlation coefficient. The formula for *r* is as in Eq. 8.

8 $$\begin{aligned} \left(r = \frac{n\sum _{}^{}{xy - (\sum _{}^{}x)(\sum _{}^{}y)}}{\sqrt{n\sum _{}^{}{x^{2} - (\sum _{}^{}{x)}^{2}}}\sqrt{n\sum _{}^{}{y^{2} - (\sum _{}^{}{y)}^{2}}}}\right). \end{aligned}$$

The equation of a regression line for an independent variable x and a dependent variable y is

$$[\widehat{y} = \text {mx} + b]$$

where $\widehat{y}$ is the predicted y-value for a given x-value. The slope (regression parameter) m and y-intercept b are given by Eq. (9).

9 $$\begin{aligned} \left(m = \frac{n\sum _{}^{}{xy - (\sum _{}^{}x)(\sum _{}^{}y)}}{n\sum _{}^{}x^{2} - (\sum _{}^{}{x)}^{2}}\right) \; \; and \; \; \left(b = \widehat{y} - m\widehat{y} = \ \frac{\sum _{}^{}y}{n}\ - m\frac{\sum _{}^{}x}{n}\right). \end{aligned}$$

*x* represents the faults in machine parts or faulty operation and its effect, *y* represents the failure dependent on these faults.

### Logistic regression

To predict whether faults will lead to failure (standardization DV, failure = 1, no failure = 0). The logistic regression curve relates the independent variable, *X* (shown in Fig. 2), to the mean of the DV, $P ((\widehat{y}))$ which is given in the Eq. (10):

10 $$\begin{aligned} \left(P = \frac{e^{ax + b}}{1 + e^{ax + b}}\right) \; \; or \; \; \left(P = \frac{1}{1 + e^{- (ax + b)}}\right), \end{aligned}$$

where *P* is the probability (the mean of *Y*) of a 1, $e = 2.7182818$, and *a* is a regression parameter represents amount of increment on the variable *y* when *x* which increases by single unit, value of b yields *P* when *X* is zero. Odds ratio for occurs failure constituents categories. We are going to study a dependent variable that is binary a categorical variable that has two values such as “occurs” and “not occurs” rather than continuous. At binominal logistic regression by knowing the reference of machine parameters and we can predict whether that fault will be cause of failure or not. We can talk about the probability and about the odds of being failure or not. For example, let’s probability of failure occurs is 0.80. Then the odds of being failure would be given from the Eq. (11).

11 $$\begin{aligned} (\text {odds} = \frac{P}{1 - P})=\bigg (odds = \frac{\text {probability of failuer}}{\text {probability of non failure}}\bigg ). \end{aligned}$$

Clearly, the probability is not the same as the odds, the odds of failure should be the opposite of the odds of non-failure. In our example, the odds of non-failure would be 0.2/0.8 or 2/8 or 0.2. We can calculate the natural logarithm, *ln* where the natural log of 8/2 is 2.217 $(ln (0.8/0.2) =2.217)$. The natural *logof*2/8 is $-\,2.217$ $(ln(.02/0.8) = -\,2.217)$, so the log odds of failure is opposite to the log odds of non-failure. The natural $log = 0$ when $X=1$, When $X > 1$, the log curves up. When *X* < 1, the natural log is less than zero, and decreases rapidly as *X* approaches zero. When $P = 0.50$, the odds are 0.50/0.50 or 1, and $ln (1) =0$. If P > 0.50, $ln(P/(1-P)$ is positive; if P< 0.50, *ln*(*odds*) is negative. *ln* logistic regression, the dependent variable is the natural log of the odds, see Eq. (12),

12 $$\begin{aligned} \left(\log \left( \text {odds} \right) = ln(\frac{P}{1 - P})\right). \end{aligned}$$

The odds are the ratio chance of occurrence of failure.

### Platform and data analysis

It is important to place the sensors in the places that accurately represent the state of the art. As an application of the above, we designed a program using Visual Basic language that collects all available data in real time from all sensors of the factory. The data acquisition system includes the entered monitoring data, checklist data, operation parameters, and sensors data. These data are processed inside the factory local network. This data analysis can be useful in the maintenance program for the machine to minimize halting time for maintenance and reduce the malfunctions during operation. The data can be classified into three categories

- *Status data* which represent the normal operation readings.
- *Caution data* the data which approach the threshold limits need preventive action.
- *Warning data* the out of limits data which need immediate action to avoid damage.

**Figure 6:**
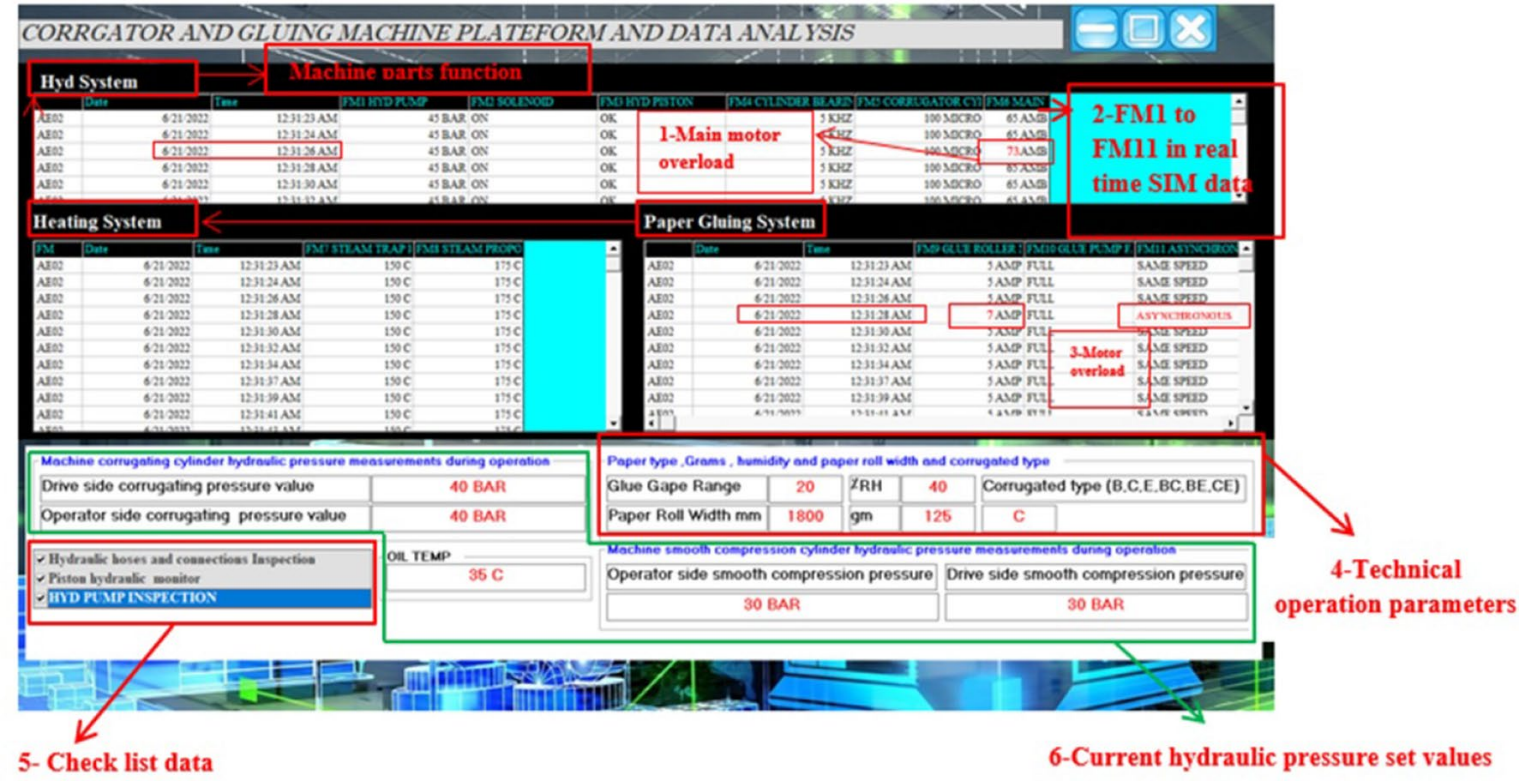
Corrugator and gluing machine platform and data analysis.


The data recorded via server to be able to build up history for each machine then, this information is sent to the cloud for use in industrial IoT applications that can be used to predict possible failure modes. Figure 6 shows the proposed model of the machine data analysis program. The pseudo code of the software is shown in algorithm (1).

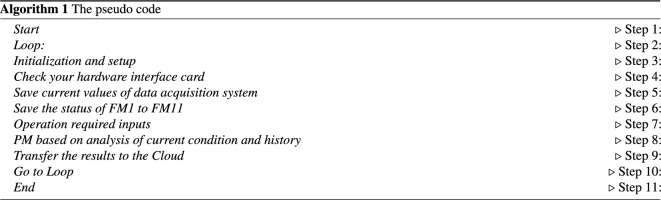


### Deep learning based fault diagnosis (DLFD)

**Figure 7:**
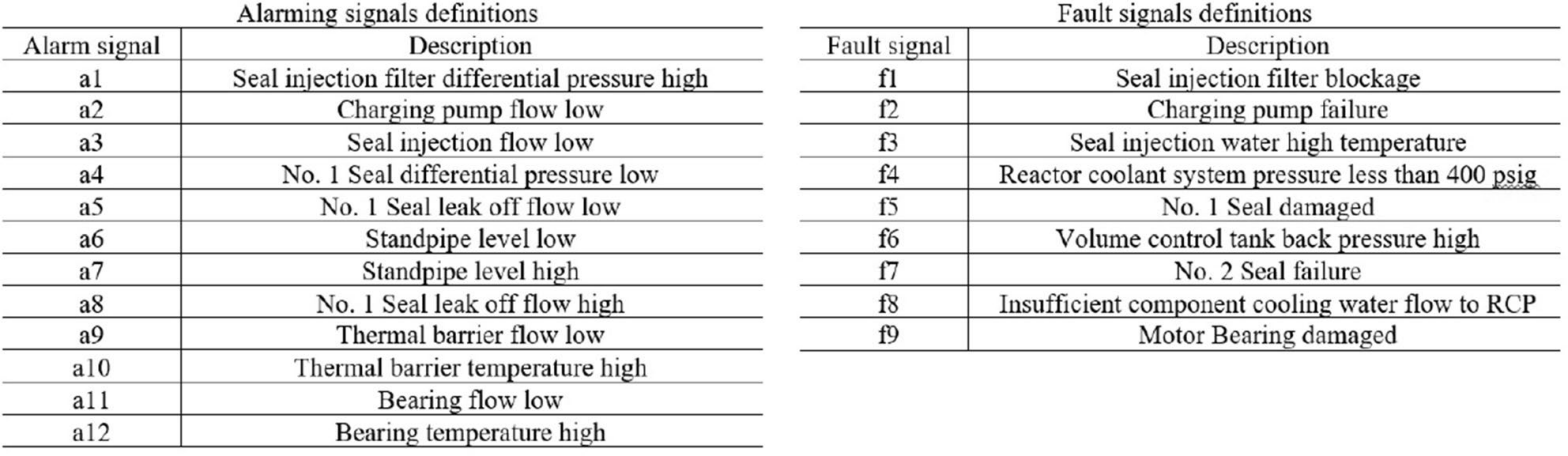
Alarming and fault definitions.

**Figure 8:**
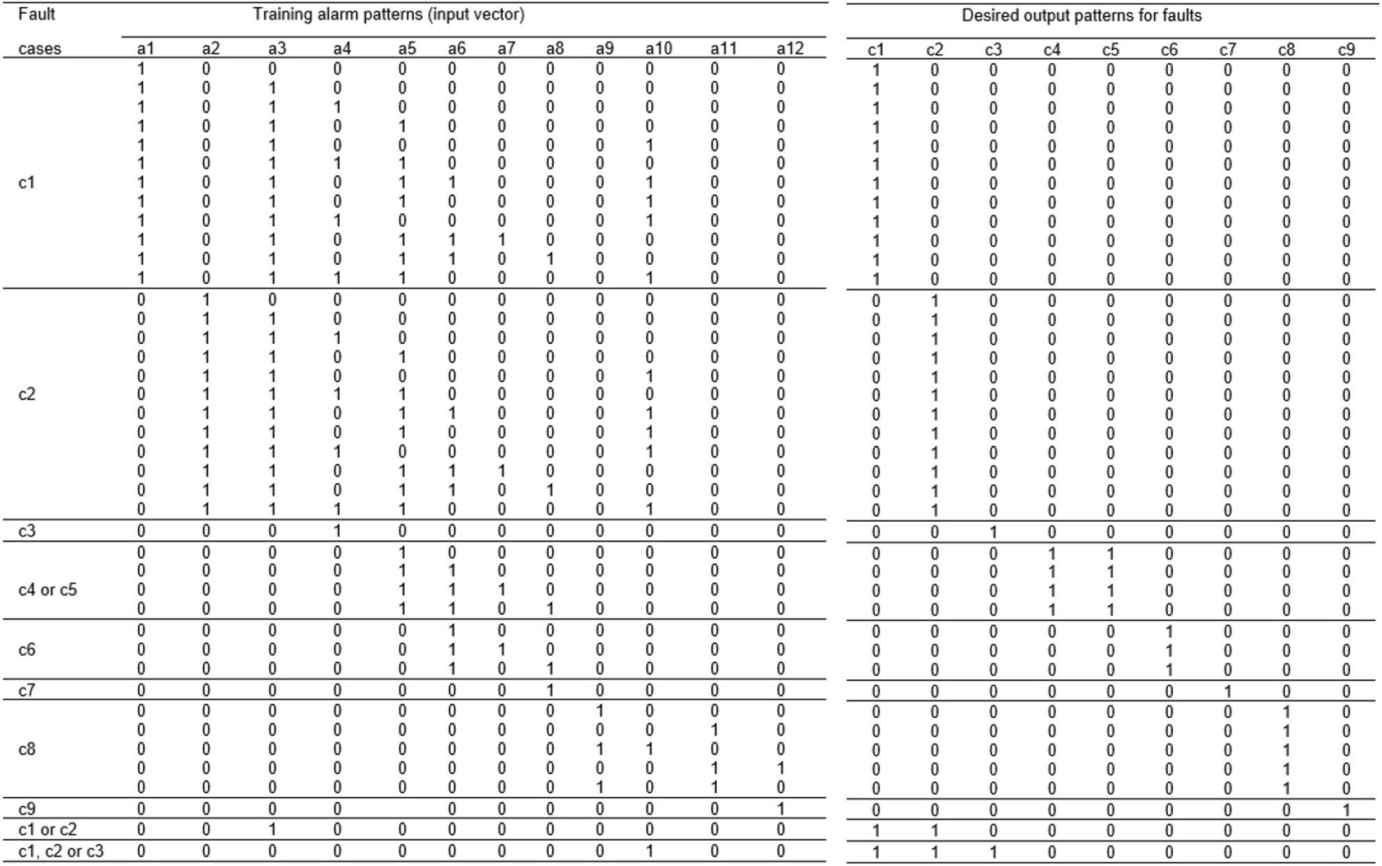
DLFD alarming (12 inputs) Faults (9 outputs) training patterns.

This section introduces more details on DLFD in a certain node, it is the cooling pump. System Cooling Pump (SCP). SCP is considered one of the most important parts of the production line (1 of 17 critical points). This critical point has 12 alarming input signals (a1, a2, a3, ...., a12) and the possibility of output faults are 9 faults (f1, f2, f3, .... f9). The definition of the faults and their corresponding alarms are shown in Fig. 7. Deep Learning pattern recognition tool in Python is used to design this ANN. It consists of 12 input nodes, 10 hidden nodes, and 9 output nodes. Different designs with different number of hidden nodes have been proposed but give great errors that ensured that using 10 hidden nodes give more accurate design with small, accepted error. As the error back propagation training algorithm (EBPTA) is running, weights of the DLFD are changing till the allowed RMS error reaches its recommended learning value, and thus learning stops. DLFD training has been achieved using 41 known alarm patterns, see Fig. 8. Then by using these weights of the DLFD, the diagnosis of any fault caused by any other alarm patterns can be achieved. For more Details, see^41^.

This proposed technique can be used in the global fault diagnosis system in all critical points in the production lines. According to every Training pattern the hidden layer nodes have their own outputs which are used as inputs of the output layer nodes. As the learning process is continuing the Root Mean Square (RMS) error decreases tell the allowed error is achieved, then the training stops, see algorithm 2. After network training every pattern of data have their own error value which is called pattern error. For More Information , please, read^41–44^.
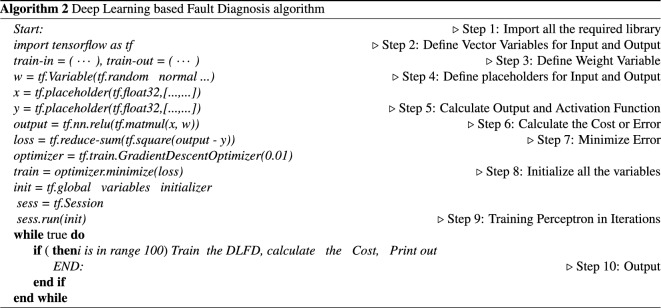


## Conclusions

The proposed maintenance analysis model is a superior analysis method since it can correlate maintenance reports of machine failures with data sent from sensors. Moreover, it can analyze the severity and evaluate the risk when data are very often imprecise and fuzzy. The traditional model of failures evaluation indicates failures FM10, FM8 and FM7 which have the highest priority and needing early corrective actions more than the rest of the failures. The proposed fuzzy FMEA model using MULTIMOORA method easily quantifies these types of data. This method includes an effective method to weight the risk factors and to rank the failure modes as a result, failures FM4, FM10, and FM6 become the highest risk priority. The results of comparison analysis show that a more accurate ranking can be determined for the machine failures by application of fuzzy set theory and MULTIMOORA method to FMEA. That prevents the machine parts to fail by which we can identify the main and sub-causes for the failure and we can determine the risk priority of failures. On the other hand the DLFD could translate the the different alarming patterns into clear faults by which it guides the control room engineers to find the faults.

## Data Availability

The Data of the submitted article are available per request to the corresponding author.

## References

[1] Ran, Y., Zhou, X., Lin, P., Wen, Y. & Deng, R. A survey of predictive maintenance: Systems, purposes and approaches. *IEEE Communications Surveys & Tutorials* 1–36 (2019).

[2] Namuduri VSPDLB, Narayanan BN, Bhansali S (2020). Review deep learning methods for sensor based predictive maintenance and future perspectives for electrochemical sensors. J. Electrochem. Soc..

[3] Yang Z, Djurdjanovic D, Ni J (2008). Maintenance scheduling in manufacturing systems based on predicted machine degradation. J. Intell. Manuf..

[4] Ayvaz S, Alpay K (2021). Predictive maintenance system for production lines in manufacturing: A machine learning approach using iot data in real-time. Expert Syst. Appl..

[5] Fortmann, I. H. & Benlian, A. *Management for Professionals, Chap. Navigating Through Digital Transformation Using Bimodal IT: How Changing IT Organizations Facilitates the Digital Transformation Journey* 393–410 (Deutsche Bahn Vertrieb GmbH, 2019).

[6] Haris STP, AsAdi M, Montreano D (2021). Machine maintenance planning in manufacturing company using rcm II methods. J. Phys. Conf. Ser..

[7] Lolli MP, Coruzzolo AM, Sgarbossa F (2021). Age-based preventive maintenance with multiple printing options. Int. J. Prod. Econ..

[8] Yang H, Li W, Wang B (2021). Joint optimization of preventive maintenance and production scheduling for multi-state production systems based on reinforcement learning. Reliab. Eng. Syst. Saf..

[9] Neto AA, Carrijo BS, Brock JGR, Deschamps F, de Lima EP (2021). Digital twin-driven decision support system for opportunistic preventive maintenance scheduling in manufacturing. Procedia Manuf..

[10] Hadi S, Gustopo D, Indra D (2020). Predictive maintenance analysis overhead crane machine in Pt bromo steel Indonesia. J. Phys. Conf. Ser..

[11] Zhang RY, Wang P, Gao RX (2018). Deep learning for improved system remaining life prediction. Procedia CIRP.

[12] Sheng YLAIA, Guo A, Peng GD (2011). Optimizing the data acquisition rate for a remotely controllable structural monitoring system with parallel operation and self-adaptive sampling. Smart Mater. Struct..

[13] Rabatel SB, Poncelet P (2011). Anomaly detection in monitoring sensor data for preventive maintenance. Expert Syst. Appl..

[14] Huang JA, Chang Q, Xiao G (2019). A maintenance and energy saving joint control scheme for sustainable manufacturing systems. Procedia CIRP.

[15] Miao JL, Zhao M, Xu X (2016). Sparse maximum harmonics-to-noise-ratio deconvolution for weak fault signature detection in bearings. Meas. Sci. Technol..

[16] Verhagen WJC, Boer LWMD (2018). Predictive maintenance for aircraft components using proportional hazard models. J. Ind. Inf. Integr..

[17] Poganik JD, Tavar J (2017). Aircraft fault forecasting at maintenance service on the basis of historic data and aircraft parameters. Eksploat. i Niezawodn..

[18] Baleentis TB, Brauers WKM (2012). Multimoora-fg: A multi-objective decision making method for linguistic reasoning with an application to personnel selection. Informatics.

[19] Najwa, N. F. The fmea traditional modifications (fmea improvement) in it risk assessment. In *Proc. Int. Appl. Bus. Eng. Conf.* 3946 (2022).

[20] Liu HC, You JX, Li P, Su Q (2016). Failure mode and effect analysis under uncertainty: An integrated multiple criteria decision making approach. IEEE Trans. Reliab..

[21] Mourtzis D (2019). Simulation in the design and operation of manufacturing systems: State of the art and new trends. Int. J. Prod. Res..

[22] Filz M-A, Langner J, Herrmann C, Thiede S (2021). Data-driven failure mode and effect analysis (fmea) to enhance maintenance planning. Comput. Ind..

[23] Mourtzis D, Angelopoulos J, Panopoulos N (2021). Collaborative manufacturing design: A mixed reality and cloud-based framework for part design. Procedia CIRP.

[24] Mourtzis D, Milas N, Athinaios N (2018). Towards machine shop 4.0: A general machine model for cnc machine-tools through opc-ua. Procedia CIRP.

[25] Ayatollahi, I., Kittl, B., Pauker, F. & Hackhofer, M. *Prototype opc ua Server for Remote Control of Machine Tools* 73–76 (2013).

[26] Liu C, Vengayil H, Lu Y, Xu X (2019). A cyber-physical machine tools platform using opc ua and mtconnect. J. Manuf. Syst..

[27] Schmid J (2021). Acquisition of machine tool data via the open source implementation open62541 for opc-ua. Procedia CIRP.

[28] Eswaran M, Bahubalendruni MVAR (2022). Challenges and opportunities on ar/vr technologies for manufacturing systems in the context of industry 4.0: A state of the art review. J. Manuf. Syst..

[29] Xiong Y, Tang Y, Zhou Q, Ma Y, Rosen D (2022). Intelligent additive manufacturing and design: State of the art and future perspectives. Addit. Manuf..

[30] Liu Y-Y, Chang K-H, Chen Y-Y (2023). Simultaneous predictive maintenance and inventory policy in a continuously monitoring system using simulation optimization. Comput. Oper. Res..

[31] van Dinter R, Tekinerdogan B, Catal C (2022). Predictive maintenance using digital twins: A systematic literature review. Inf. Softw. Technol..

[32] Gupta V, Mitra R, Koenig F, Kumar M, Tiwari MK (2023). Predictive maintenance of baggage handling conveyors using iot. Comput. Ind. Eng..

[33] Liu C (2022). Probing an intelligent predictive maintenance approach with deep learning and augmented reality for machine tools in iot-enabled manufacturing. Robot. Comput.-Integr. Manuf..

[34] Brauers WKM, Zavadskas EK (2006). The moora method and its application to privatization in a transition economy. Control. Cybern..

[35] Brauers W, Zavadskas EK (2010). Project management by multimoora as an instrument for transition economies. Technol. Econ. Dev. Econ..

[36] Baskar C, Parameshwaran R, Nithyavathy N (2020). Implementation of fuzzy-based integrated framework for sesame seed separator development. Soft. Comput..

[37] Zhang C, Chen C, Streimikiene D, Balezentis T (2019). Intuitionistic fuzzy multimoora approach for multi-criteria assessment of the energy storage technologies. Appl. Soft Comput. J..

[38] Liu HC, Fan XJ, Li P, Chen YZ (2014). Evaluating the risk of failure modes with extended multimoora method under fuzzy environment. Eng. Appl. Artif. Intell..

[39] Liu, H. C. *Part I: FMEA and Its Improvements* (Book Ref, 2016).

[40] Chang DS, Chung JH, Sun KL, Yang FC (2012). A novel approach for evaluating the risk of health care failure modes. J. Med. Syst..

[41] Metwally, M. A., Aboshosha, A., Khalil Ibrahim, D. & EL-Zahab, E. E.-D. A. Applying neurofuzzy computing for safety improvement of nuclear power reactor. In *Proc. 14th International Middle East Power Systems Conference (MEPCON10)* (Cairo University, 2010).

[42] Aboshosha, A. *Using Neural Networks in Control and Fault Diagnosis of Nuclear Plants*. Mater’s thesis, Menoufia University, Faculty of Electronics (1997).

[43] Aboshosha, A. Neurofuzzy computing aided fault diagnosis of nuclear power reactors. In *Proc. 7th ICEENG Conference, Military Technical College, Cairo, Egypt*. 10.13140/2.1.3799.8722 (2010).

[44] Aboshosha, A. *et al*. Using neural networks in fault diagnosis of nuclear power reactor. In *The Fourth IEEE International conference, Electronics, Circuits, and systems ICECS 97, Cairo, Egypt* (1997).

